# Retirement, happiness, and health in Japan

**DOI:** 10.1093/geroni/igab046.3288

**Published:** 2021-12-17

**Authors:** Shohei Okamoto, Erika Kobayashi

**Affiliations:** Tokyo Metropolitan Institute of Gerontology, Itabashi-ku, Tokyo, Japan

## Abstract

While health effects of retirement have been well studied so far, previous findings remain inconclusive, and mechanisms underlying the linkage between retirement and health are unclear. This can be driven by regional or cohort heterogeneity as well as methodological differences, such as outcome measures and identification strategies; thus, much evidence needs to be accumulated. Utilising a national household survey conducted every year in 2004-2019 in Japan (the Japan Household Panel Survey), we evaluate the effects of retirement among Japanese adults aged 50-75 on their happiness and health in addition to other outcomes that could attribute to happiness or health changes (e.g. health behaviours, time use for some activities, and the expenses by item). As outcomes are not measured every year, we analyse 4,340-7,902 person-year observations by 756-1,389 individuals with the necessary information from 2009. To deal with the potential endogeneity of retirement, we adopt an instrumental variable approach utilising changes in retirement policy and public pension eligible age. Consequently, instruments seem valid only for men, and we find that retirement increases male retirees’ happiness and decreases psychological stress while effects on other health measures are not observed. Although their satisfaction with their income decline, perhaps because of the loss of their wage income, they tend to increase the proportion of expenses for cultural and recreational activities. Enhancement in personal life quality by more leisure activities and stress reduction from work, rather than improvements in health behaviours and physical health, may be key to understanding health benefits in retirement.

